# Children of Alcoholics Foundation

**Published:** 1997

**Authors:** Migs Woodside

**Affiliations:** Migs Woodside is the founder and former president of the Children of Alcoholics Foundation in New York City and a member of the Board of Directors of Phoenix House, of which the Foundation is an affiliate

**Keywords:** children of alcoholics, organization, familial alcoholism, prevention through information dissemination, prevention through education, education and training, curriculum, school based prevention, prevention program, health care worker, workplace based prevention, outreach

In 1982 the Nation’s first comprehensive report on children of alcoholics (COA’s) was prepared upon request as part of the New York State Heroin and Alcohol Abuse Study for New York State Governor Hugh L. Carey and Special Counselor on Alcoholism and Drug Abuse Joseph A. Califano, Jr. The report addressed the status of and critical gaps in alcohol-related research, existing programs and barriers to service delivery, the need to educate and train professionals, and the need for legislation. It also identified strategies to help this high-risk group break the cycle of family addiction.

Upon release, the report received nationwide media attention, including front-page coverage by *The New York Times*. Subsequently, an avalanche of telephone calls and letters poured into the Study’s offices from across the United States and Western Europe from COA’s seeking help for themselves and from parents seeking help for their children. In response to public demand, a group of professionals, scientists, celebrities, and corporate leaders established the Children of Alcoholics Foundation. Specifically designed to meet the needs of COA’s, the organization has mounted innovative programs and developed a collection of useful materials to assist this vulnerable group.

## Meeting the Needs of COA’s

Based on a child-centered definition of COA, the Foundation defines alcoholism as the child’s perception that a parent drinks too much and that the drinking interferes with the child’s life.

This definition underscores the Foundation’s underlying philosophy: COA’s may need and deserve help, whether or not their parent or parents continue to drink, and that for some, help may be needed over a lifetime. Some COA’s require counseling or long-term treatment; others may do well, depending on their level of vulnerability. Nonetheless, coping behaviors employed effectively in childhood may become self-defeating in adulthood. Consequently, all COA’s need to be educated on the effects of living with an alcohol-abusing parent.

Rather than promote new bureaucracies and systems, the Foundation advocates an efficient, cost-effective strategy: It works within existing structures and targets people already in contact with COA’s who can potentially help improve their lives but are currently unaware of the inherited predisposition to alcoholism and the pain and problems caused by living with an alcoholic parent.

## Fulfilling the Foundation’s Goals

To meet its goals (i.e., promote wellness, prevent future addiction, and help COA’s reach their full potential), the Foundation holds conferences, prepares reports, and disseminates research findings. It also has produced numerous research-based instructional materials, educational curricula, and training programs — for use by teachers, physicians, guidance counselors, mental health and addiction specialists, employers, scientists, and the general public — that translate research findings into practical skills and knowledge. Each of these components is discussed below in detail.

### Promoting Research

Developing and disseminating research findings and applying this knowledge to COA-focused programs have always been fundamental to the Foundation’s mission. Through meetings and forums aimed at identifying and addressing COA research needs, the Foundation has developed strong ties with the scientific community.

Through these meetings, the Foundation has created an agenda of COA research topics that includes genetic markers, prevention of fetal alcohol syndrome and fetal alcohol effects, the effects of identifying and labeling COA’s in various systems, social services system utilization, screening tools to match COA’s with appropriate programs, and evaluation of program effectiveness. The Foundation hosted the initial meeting of what later became known as the Collaborative Study on the Genetics of Alcoholism (COGA) and continues its involvement through participation on COGA’s Committee on Ethics. The Foundation also maintains close ties with the research community through its Scientific Advisory Committee.

### Producing and Disseminating Reports

The Foundation produces reports that contain specific recommendations and action plans and distributes them to professionals, service providers, scientists, government leaders, and the media. The latter publications include, for example, reports on adolescent responses to televised beer advertisements, the problems of inner-city children of addicted parents, the impact of fetal alcohol syndrome on children’s ability, as well as the outcome of a survey of Fortune 500 corporations on their level of knowledge of and assistance to adult COA’s in the workplace.

### Promoting School-Based Programs

The Foundation’s school-based initiative includes several components, including “The Images Within, A Child’s View of Parental Alcoholism,” a three-session program for children ages 10–13. This program assists youngsters whose parents abuse alcohol; educates the children’s friends on ways to help; and features a teacher’s guide, brochures, quizzes, and drawings by COA’s.

The Foundation also produced a series of videos, accompanied by discussion guides, as teaching aids for teachers and addiction specialists who work with children. These films feature COA’s who have received counseling and who talk candidly about their feelings, experiences, and coping techniques. In addition, the Foundation has recently published a manual to guide school professionals without mental health backgrounds in leading support groups for children of alcoholics and other substance abusers.

Another Foundation-sponsored, school-based program targets school nurses, who are often the first people to recognize COA’s because of their increased physical problems, such as insomnia, stomachaches, headaches, and bruises. To assist nurses in working with these children and to dispel myths about family alcoholism, the Foundation developed a workbook for nurses to use to encourage children to discuss their feelings and help them develop self-esteem. In addition, the Foundation created a poster for children that advocates school nurses as a safe and positive resource.

The Foundation’s new on-campus school program, “Options: Mastering the Challenges of College Life,” targets college students, many of whom may be from alcohol-abusing families and may be exposed to risky behaviors in school. The program’s three components include a handbook for students from substance-abusing families; awareness-building materials for all students (i.e., booklets, posters, and wallet cards); and a training curricula for counselors, prevention specialists, medical and health care professionals, chaplains, and residential and student life professionals.

### Reaching Medical and Health Care Professionals

The Foundation’s initiatives for medical and health care professionals draw heavily from research findings and the organization’s long-term relationship with the American Academy of Pediatrics. In conjunction with the Academy, the Foundation surveyed the Academy’s members to determine those subjects that pediatricians have the most difficulty discussing with patients and their families. Results indicated that the most difficult topics are parental substance abuse, parental sexual abuse of children, and adolescent drinking. In response, the Foundation created “Opening and Closing Pandora’s Box” to help physicians and other child health care providers talk about these issues with children and their families.

The Foundation also developed a video and workbook for physicians to use to help them identify COA’s and refer them to local resources. The material is used in medical schools, hospitals, and private practices.

In addition, to help mental health and other behavioral health care professionals promote wellness in their work with adult COA’s and break the intergenerational cycle of this disease, the Foundation developed the parenting program “Discovering Normal,” a six-session training manual designed to teach parents more effective parenting skills. The program is used in mental health clinics, in social services agencies, on Native American reservations and military bases across the Nation, and in many foreign countries.

The Foundation is also concerned about the effects of managed care on access to service and the nature of service delivery to youngsters from alcoholic families. Its preliminary survey of programs for young COA’s in New York State found that 25 percent were denied services because of a lack of education and training of managed care specialists about the effects of parental alcohol abuse on children’s physical, mental, and emotional health as well as misconceptions about the insurance law, including regulations and their implementation. The Foundation currently serves on a council that is preparing a technical assistance guide to address these obstacles to service delivery.

### Providing Information in the Workplace

Although early intervention in childhood is key to preventing future addiction problems in adulthood, the Foundation has also included the workplace within its scope of activities. The Foundation’s survey of major corporations showed that medical directors and human resource specialists failed to understand that the long-term effects from family alcoholism could cause problems on the job. Although many adult COA’s are productive employees, even those who do not have addiction or mental health problems can benefit from education about the disease and its ramifications. To this end, the Foundation has displayed an exhibition of 500 drawings, stories, and poems in various corporate headquarters, prompting some employees to discuss, for the first time, past or present family addiction with human resource and employee assistance program (EAP) personnel.

Furthermore, in addition to producing reports for businesses, the Foundation has developed tools to assist managers, supervisors, EAP personnel, and employees in the workplace. A five-module curriculum, “Survival Skills for the Work-place,” presents methods for improving understanding and performance. Topics include time management, stress management, effective listening, assertive communication, and change management. A companion video, “Working It Out,” provides EAP directors with a nonthreatening way to introduce the topic of parental substance abuse.

## Additional Initiatives

The Children of Alcoholics Foundation also conducts a number of other initiatives. For example, in an effort to reach a variety of audiences, the Foundation sponsors a dramatic portrayal of alcoholism’s effects on family members. Incorporating an audience discussion led by a trained professional, the play is used in a multitude of settings, from youth groups to schools and for the general public by insurance companies and corporations.

In addition, during 1997 the Foundation became affiliated with Phoenix House, an agency for substance abusers that has facilities in New York, New Jersey, Texas, and California. The relationship between the organizations provides an essential link with front-line treatment professionals while focusing on the reality of polyaddiction.

Furthermore, in order to form a new alcoholism constituency, the Foundation has conducted an investigation to find out what people are willing to do to raise public awareness and the actions they will take to support increased attention and resources for alcoholics and their children. Despite the stigma and denial associated with the disease, many people affected by alcoholism are coming forward to speak at schools and treatment centers, lobby Government officials for increased funds, and pressure the media to portray addiction and its effects more realistically.

Since its inception, the Children of Alcoholics Foundation has worked hard to meet the needs of a vulnerable and often forgotten group within the population. Through the organization’s dedication and commitment, its dissemination of research-based information, its development of educational publications and programs, and its direct assistance efforts, the Children of Alcoholics Foundation continues to improve the lives of both young and adult COA’s.

For further information, contact Children of Alcoholics Foundation at 212–757–2100, ext. 6370, or to access the HelpLink telephone line, call 1–800–359–COAF (2623).

